# Maximizing the diagnostic information from biopsies in chronic inflammatory bowel diseases: recommendations from the Erlangen International Consensus Conference on Inflammatory Bowel Diseases and presentation of the IBD-DCA score as a proposal for a new index for histologic activity assessment in ulcerative colitis and Crohn’s disease

**DOI:** 10.1007/s00428-020-02982-7

**Published:** 2020-12-29

**Authors:** Corinna Lang-Schwarz, Abbas Agaimy, Raja Atreya, Christoph Becker, Silvio Danese, Jean-François Fléjou, Nikolaus Gaßler, Heike I. Grabsch, Arndt Hartmann, Kateřina Kamarádová, Anja A. Kühl, Gregory Y. Lauwers, Alessandro Lugli, Iris Nagtegaal, Markus F. Neurath, Georg Oberhuber, Laurent Peyrin-Biroulet, Timo Rath, Robert Riddell, Carlos A. Rubio, Kieran Sheahan, Herbert Tilg, Vincenzo Villanacci, Maria Westerhoff, Michael Vieth

**Affiliations:** 1 Institute of Pathology, Klinikum Bayreuth GmbH, Preuschwitzer Str. 101, 95445 Bayreuth, Germany; 2 Institute of Pathology, Friedrich-Alexander University, Erlangen, Germany; 3 Medical Clinic 1, Department of Medicine & Deutsches Zentrum Immuntherapie DZI, University Hospital, Friedrich-Alexander University, Erlangen, Germany; 4 The Transregio 241 IBDome Consortium, Erlangen, Germany; 5 Department of Gastroenterology, IBD Centre, Humanitas Research Hospital, Via A. Manzoni 56, 20089 Rozzano, Milan, Italy; 6 Pathology Department, Saint-Antoine Hospital, APHP, Sorbonne University, Paris, France; 7 Institute for Legal Medicine, Section Pathology, University Hospital, Jena, Germany; 8 Department of Pathology, GROW - School for Oncology and Developmental Biology, Maastricht University Medical Center, Maastricht, The Netherlands; 9 Pathology and Data Analytics, Leeds Institute of Medical Research at St James’s, University of Leeds, Leeds, UK; 10 The Fingerland Department of Pathology, Faculty of Medicine and University Hospital, Charles University, Hradec Králové, Czech Republic; 11 Charité-Universitätsmedizin Berlin, Corporate Member of Freie Universität Berlin, Humboldt-Universität zu Berlin and Berlin Institute of Health, iPATH.Berlin, Campus Benjamin Franklin, Hindenburgdamm 30, 12200 Berlin, Germany; 12 Department of Pathology, Moffitt Cancer Center, Tampa, FL USA; 13 Institute of Pathology, University of Bern, Bern, Switzerland; 14 Department of Pathology, Radboud University Medical Centre, Nijmegen, The Netherlands; 15 INNPATH, Institute of Pathology, Tirol Kliniken, Innsbruck, Austria & Patho im Zentrum, St. Pölten, Austria; 16 Department of Gastroenterology, Nancy University Hospital, Vandoeuvre, France & Inserm U1256, Lorraine University, Vandoeuvre, France; 17 Pathology and Laboratory Medicine, Mount Sinai Hospital, University of Toronto, Toronto, ON Canada; 18 Department of Pathology, Karolinska Institute and University Hospital, Stockholm, Sweden; 19 Department of Pathology & Centre for Colorectal Disease, St Vincent’s University Hospital & University College, Dublin, Ireland; 20 Department of Internal Medicine I, Gastroenterology, Hepatology, Endocrinology and Metabolism, Medical University Innsbruck, Innsbruck, Austria; 21 Department of Pathology, Spedali Civili di Brescia, Brescia, Italy; 22 Department of Pathology, University of Michigan, Ann Arbor, MI USA

## Introduction

Chronic idiopathic inflammatory bowel disease (IBD) cases are on the rise, with approximately 6.8 million people diagnosed in 2017 [1]. Diagnosing the two main forms of IBD, ulcerative colitis (UC) and Crohn’s disease (CD), involves a combination of clinical history, laboratory findings, imaging, endoscopy and histology [2].

The histopathological diagnosis of IBD is based on a combination of microscopic findings, with consideration of clinical data that include the patient’s age, symptoms, duration of symptoms and endoscopic results [3–17].

CD is a chronic, progressively destructive disease with an intermittent course in most cases. UC is most often described as relapsing and remitting, with symptoms of active disease that alternate with periods of clinical quiescence called remission [18, 19]. Until recently, therapeutic strategies in both diseases aimed to achieve adequate control of gastrointestinal inflammation. In recent years, however, mucosal healing has become the key treatment goal in IBD. It is associated with improved clinical outcomes, prolonged remission, fewer hospitalizations and decreased probability for surgery [20–36]. Patients with mucosal healing have lower rates of clinical relapse compared to those with evidence of active or quiescent disease. Endoscopic evaluation without histology, however, may be insufficient to deem treated IBD mucosa as completely healed [34, 36]. Furthermore, histologic identification of active disease has also been shown to better predict the development of neoplasia in patients with UC compared to endoscopic assessment of activity [30–32, 34, 37]. The fact that concordance between endoscopic and histological remission is moderate only—with histological remission being superior—underscores the necessity of incorporating histologic methods of activity scoring into clinical trials [34]. In fact, in 2016, the Food and Drug Administration (FDA) of the United States Department of Health and Human Services recommended that histologic assessment be used in conjunction with endoscopy [38].

A number of histologic indices for assessing activity in UC and CD exist. Nevertheless, they all have limitations, including lack of full validation, difficulty of usage and heterogeneity in their standards for distinguishing between quiescent disease and histologic normalization [39, 40]. Moreover, there is also a need for standardization of biopsy procedures in UC and CD on the endoscopic end.

Recommendations for biopsy procedures and IBD scoring exist from many internationally recognized organizations, such as the European Crohn’s and Colitis Organisation (ECCO), the World Gastroenterology Organisation (WGO), the British Society of Gastroenterology (BSG), the International Organization for the Study of Inflammatory Bowel Diseases (IOIBD), the American College of Gastroenterology (ACG) and the American Society for Gastrointestinal Endoscopy (ASGE). Some of them have recently been updated [17, 19, 28, 41–51].

However, in practice, these existing recommendations are not widely adhered to and there is still a need for standardizing the number of IBD biopsies, the locations they are taken from and an accepted validated IBD histologic assessment tool for daily practice.

For these reasons, the objectives of the 2020 International Consensus Conference on IBD Activity Scoring were (i) to review existing recommendations and to agree upon ten recommendations with the highest impact to maximize diagnostic information from biopsies for UC and CD and (ii) to agree on a simple, histologic scoring system for both types of IBD that is able to distinguish between quiescent disease and mucosal healing and has potential for use in daily routine practice as well as for clinical trials.

## Material and methods

### Consensus process

The meeting took place from the 8th to the 10th of January 2020 at the Institute of Pathology of the Friedrich-Alexander University in Erlangen, Germany, with participants from 12 countries. Six sessions comprising presentations, discussions and group microscopy sessions took place. The conference was followed by a validation process of the consensus-approved scoring index. This involved individual member evaluations of digitally scanned IBD biopsy slides.

### Steering committee and participants

The steering committee (MN, AH and the chairman MV) organized the meeting in Erlangen. Twenty-seven participants with expertise in IBD from the USA, Canada and Europe were invited to attend the face-to-face meeting, and 25 agreed to participate (17 gastrointestinal pathologists, 6 gastroenterologists and two translational researchers). Five were unable to attend but participated in the post-meeting validation of the agreed-upon scoring system as well as the voting at final ballot according to a modified Delphi panel. All participants were voting members.

### Methodological exactness

#### Search strategy and sources of evidence

The recommendations that reached consensus were based on peer-reviewed publications in addition to expert opinions, accepted practice standards and consensus. To obtain consensus, a two-round modified Delphi process was used. For this purpose, two rounds of systematic literature searches regarding IBD and activity scoring on PubMed, the Cochrane Library and EMBASE were performed. The first took place prior to the meeting and was circulated for review to all of the participants. At the end of the face-to-face meeting, a first voting round on the developed statements took place. Additional literature from a second literature search was added post-meeting using combinations of key words such as “IBD”, “UC”, “CD”, “histopathology” and “activity”. Six months post-meeting, a final anonymous Delphi questionnaire round took place. Participants were asked to review and reconsider their initial ratings for the developed statements on a circulated questionnaire using a 5-point Likert scale (5 = agree (100%), 4 = partly agree (75%), 3 = neutral (50%), 2 = partly disagree (25%), 1 = disagree (0%)) [41, 42]. Strength of consensus was graded as follows: < 75%, no consensus; 75–95%, consensus; and > 95%, strong consensus.

#### Evidence levels and recommendation grades

The literature was rated according to the evidence classification of the Oxford Centre for Evidence-based Medicine (OCEBM), using the 2011 version (supplemental Table 1) [52].

**Table 1:** Recommendations that achieved consensus

Statement	Voting results		
	Votes at last ballot (%)	Evidence level	Recommendation grade
1. If colonoscopic biopsies are being taken for the diagnosis of chronic idiopathic IBD, the samples should be sent in separate, designated containers, particularly biopsies of the rectum	95	1a–2b	B
2. 2 or more tissue samples from separate areas within the same bowel segment should be sent in each designated container	98	1a–2b	B
3. If Crohn’s disease is in question, additional biopsies from the upper gastrointestinal tract should also be considered	92	1b–3a	C
4. Assessing the degree of activity should be carried out on the worst affected biopsy	100	1a–2a	B
5. The information between pathologist and clinician should include core features that are relevant for the IBD diagnosis as well as for IBD activity scoring	98	1a–3b	C
6. The histological assessment should evaluate the distribution of overall findings, the presence of chronic injury and the activity of inflammation	100	1a–3b	C
7. The scoring system (the IBD-DCA score) developed by the consensus group based on the factors of distribution (D) of changes, chronic injury (C) and activity of inflammation (A) is a proposal for a standardized and user-friendly tool for histologic activity assessment	98	2b	B
8. The pathology report can include routine text as well as the IBD-DCA score as qualitative and quantitative information for clinicians	93	5	D
9. Activity scoring is ideally performed for every container separately	83	1b–3b	C
10. The IBD-DCA score is suggested for use in daily routine practice	95	5	D

Evidence and recommendations from established clinical and pathological guidelines were also taken into account, including documents from the ECCO, the ASGE, the WGO, the ACG, the IOIBD, the Updated S3-Guideline of the German Society for Digestive and Metabolic Diseases (DGVS), the updated BSG guidelines and a practical guide on tissue pathways for gastrointestinal and hepatopancreatobiliary pathology [2, 41, 43–51, 53–55].

Evidence levels (ELs) and grades of the recommendations are given below where possible. In accordance with the OCEBM, recommendation grades are defined as the following: A, consistent level 1 studies; B, consistent level 2 or 3 studies or extrapolations from level 1 studies; C, level 4 studies of extrapolations from level 2 or 3 studies; and D, level 5 evidence or troublingly consistent or inclusive studies of any level.

The recommendations, rather than being rules, are intended to help endoscopists maximize the potential diagnostic information they can render from biopsies to optimize their patients’ care. An overview of the consensus process is available as supplemental Fig. 1.

**Fig. 1:**
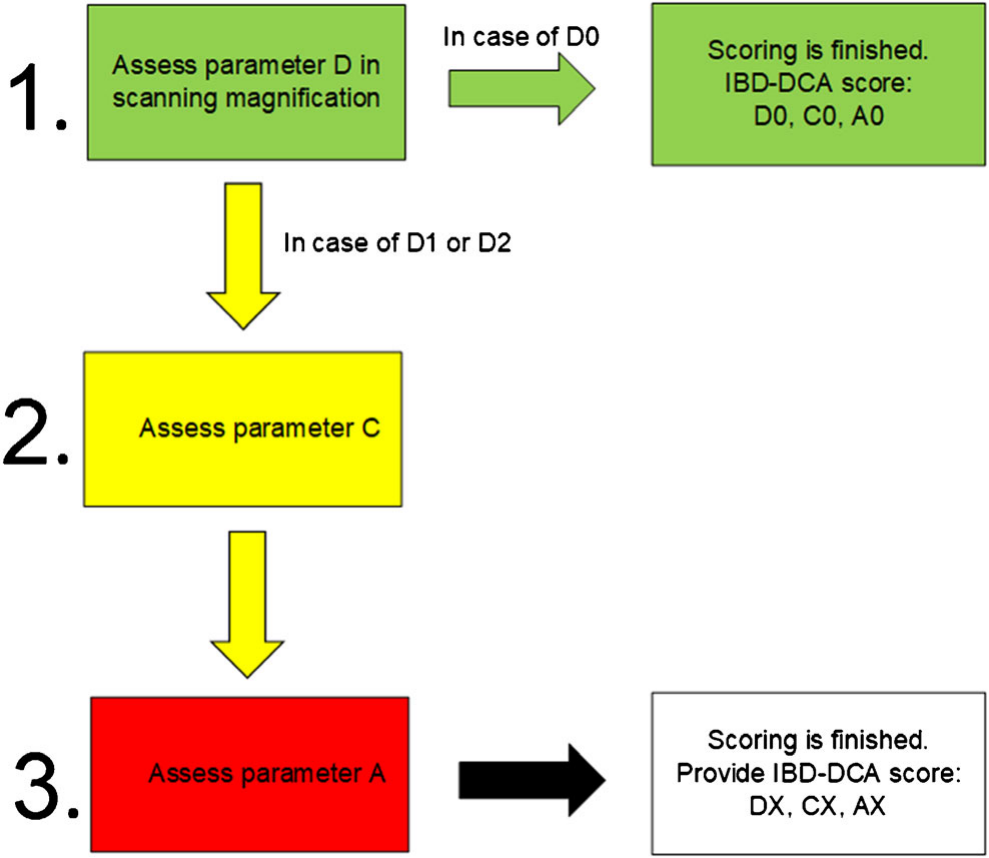
Proposed stepwise algorithmic assessment of the ID-DCA score

### Microscopy sessions

Prior to the meeting, haematoxylin and eosin (H&E)-stained slides of 59 UC large bowel biopsy cases and 25 CD biopsy sets from large bowel, terminal ileum and stomach were digitalized at the Institute of Pathology, Bayreuth, using the slide scanner NanoZoomer S360 (Hamamatsu, Herrsching am Ammersee, Germany). All participants had access to the slides prior to the meeting on a virtual, password-secured platform (software: NDP.view 2; Hamamatsu, Herrsching am Ammersee, Germany). Microscopy sessions during the face-to-face meeting allowed for group discussions of selected cases around a multi-headed microscope (Olympus BX53; Olympus, Hamburg, Germany).

### Presentations

The conference presentations comprised the scientist’s (CB), the clinician’s (MN) and the pathologist’s (MV) views concerning IBD activity scoring, along with their field-specific needs for an approved scoring system.

## Results and discussion

### Recommendations

An overview of the 10 recommendations that achieved consensus is shown in Table 1.

#### Recommendation 1

*If colonoscopic biopsies are being taken for the diagnosis of chronic idiopathic IBD, the samples should be sent in separate, designated containers, particularly biopsies of the rectum (vote: strong consensus (95%), level of evidence: 1a–2b, recommendation grade: B).*

#### Evidence and decision

When the initial diagnosis of IBD is suspected, current guidelines and consensus papers recommend an ileocolonoscopy with biopsies from the terminal ileum as well as from each colonic segment: caecum, ascending colon, transverse colon, descending colon, sigmoid and rectum [2, 19, 43, 45, 46, 56, 57]. Rather than pooling biopsies from multiple sites into the same container, the biopsies should be delivered to the pathologist in separate, designated containers. This assists in distinguishing between UC and CD and for properly evaluating the distribution of the disease [2, 4, 43, 53, 56, 58, 59] (EL 2a–2b). The importance of proper sampling and designating biopsy sites is exemplified in patients who exhibit unusual distribution of inflammation. Without clear designation of separate sites, it is challenging to diagnose CD in patients who lack terminal ileum involvement, or UC in patients who have proctitis, caecal patch and backwash ileitis. Other reasons to separately designate bowel sites are important to note. For one, some cases can be classified as UC or CD only from the course of the disease. Tracking the patients’ site-specific histology over time can help establish the diagnosis [60–65] (EL 1a–2b). Moreover, treatment may alter the distribution of inflammation and it may be useful to follow the effects of therapy for each bowel site. This can also lead to accurately establishing if histologic normalization has occurred as a result of therapy [66–73] (EL 1a–2a). Finally, designating different bowel sites can have treatment implications. Local treatment with 5-aminosalicylic acid or steroids is sufficient in most cases of UC that are restricted to the rectum. However, solitary rectal involvement may not be confirmed histologically if the patients’ rectal and colonic biopsies are pooled [71–73] (EL 1a).

#### Recommendation 2

*Two or more tissue samples from separate areas within the same bowel segment should be sent in each designated container (vote: strong consensus (98%), level of evidence: 1a–2b, recommendation grade: B).*

#### Evidence and decision

Current scoring systems have been applied to biopsy material collected under diverse protocols that include different numbers of tissue samples from different sections of the large bowel. In fact, there are protocols that allow for the bare minimum of rectum-only samples. There is clearly a need to standardize sampling protocols in clinical trials in order to optimize comparisons between treatment outcomes [20, 74] (EL 2a–2b). Our recommendation to sample a minimum of 2 biopsies from separate areas within the same bowel segment is therefore intended to standardize and optimize biopsy evaluation. This is supported by Mosli et al. [75]. They concluded that it is imperative to obtain at least 2 mucosal biopsy samples for evaluation of UC as the disease changes during the natural disease course and with local and systemic treatment [60, 74–76] (EL 1b–2a). Collecting more than one biopsy also decreases the problem of sample quality and tissue orientation issues. Additionally, as suggested by the ECCO recommendations, biopsies can be properly oriented by use of acetate strips [49, 77]. Further support for the requisite number of biopsy samples is seen in ECCO guidelines, which recommend that a minimum of two biopsies per bowel site be obtained to diagnose CD. For CD, samples should preferably be obtained from both diseased and uninvolved areas [43, 78–86] (EL 1a–2a). If samples are taken only from macroscopically or endoscopically suspicious areas, a pathologist without endoscopic information may not be able to incorporate evidence of discontinuous disease distribution to support an accurate diagnosis of CD. As such, our recommendation advises sampling from separate areas within the same bowel segment and also proper labelling of the containers to ensure that the pathologist receives proper information about the biopsies’ origin.

#### Recommendation 3

*If Crohn’s disease is in question, additional biopsies from the upper gastrointestinal tract should also be considered (vote: consensus (92%), level of evidence: 1b–3a, recommendation grade: C).*

#### Evidence and decision

Microscopic inflammation of the upper gastrointestinal (GI) tract is quite common in CD and comparatively rare in UC [87] (EL 2b). The reported frequency of upper GI tract involvement in CD varies widely, from 6.5% in a recent analysis of the Swiss IBD Cohort Study Group to 40.7% in a study of paediatric CD cases, even in the absence of specific upper gastrointestinal symptoms. As such, Castellaneta et al. [88] concluded that upper GI tract endoscopy should be part of the first-line investigation in all new cases suspected of IBD, particularly as the absence of specific upper GI symptoms does not exclude the presence of upper GI inflammation [87, 89] (EL 2b). Patches of acute inflammation of the stomach and duodenum as well as deep acute inflammation of the duodenum raise strong suspicion for CD in *Helicobacter pylori* (HP)-negative patients. In fact, the histological pattern of a “focally enhanced” gastritis in HP-negative patients with CD was first described in 1997 by Oberhuber et al. [90] (a participant of the current consensus conference). Overall, they found granulomas and/or a focally enhanced gastritis in 76% of HP-negative patients with CD and in 0.8% of controls. Moreover, there was no correlation between the presence of focally enhanced gastritis and clinical findings [90] (EL 1b). It should be noted, however, that the presence of granulomas is helpful but occurs in only 9–14.6% of upper GI biopsies [90–92] (EL 1b–3a). In summary, if CD is the diagnosis of clinical concern, the consensus group recommends additional duodenogastroscopy to help consolidate a diagnosis of CD. A minimum of 2 biopsies each from duodenum, antrum and corpus sites should be sent to the pathologist in separate containers [43, 44].

#### Recommendation 4

*Assessing the degree of activity should be carried out on the worst affected biopsy (vote: strong consensus (100%), level of evidence: 1a–2a, recommendation grade: B).*

#### Evidence and decision

Assessing the histologic activity of UC is especially important in monitoring treatment follow-up. For clinical trials, scoring is typically performed on the sample exhibiting the highest activity as this may be the most important factor in the further evolution of the disease [75] (EL 1a). It is also important to grade activity based on the most diseased biopsy fragment because it is paramount in establishing whether histologic mucosal healing has truly been achieved. The consensus participants agreed to define histologic mucosal healing as normal mucosa without any pathological changes. This includes the absence of changes seen in quiescent colitis, which encompasses architectural abnormalities as well as increased chronic inflammatory infiltrates. Therefore, grading should be done on the most affected biopsy to indicate if quiescent colitis is present, as quiescent disease is not considered equivalent to the histologic normalization required to meet criteria for histologic mucosal healing. A detailed description of the normal histology of the colon has been published by Levine and Haggitt [93] (EL 2a).

#### Recommendation 5

*The information between pathologist and clinician should include core features that are relevant for the IBD diagnosis as well as for IBD activity scoring (vote: strong consensus (98%), level of evidence: 1a–3b, recommendation grade: C).*

The following core features (or key elements) refer to details that are essential for the pathologist to be able to establish a diagnosis of IBD. They also, in turn, refer to information that the pathologist should communicate to the clinician for the patient’s management.

The core features to be communicated to the pathologist by the clinician include:

- Patient’s age [92, 94–101] (EL 1a–2b)
  In the vast majority, patients with UC and CD are usually diagnosed in their 20s and 30s, although IBD occurs at any age.
- Type and duration of symptoms [8, 10, 12, 45] (EL 2a–2b)
  This information is important given that infectious colitis is one of the main considerations in the differential diagnosis of IBD. The symptoms of infectious colitis can be similar to IBD; however, they usually last for less than 1 month. Therefore, a diagnosis of IBD is more likely with longer duration of symptoms.
- Endoscopic findings: The distribution and type of abnormalities noted on endoscopy should be relayed to the pathologist, along with the key clinical question the pathologist is being asked to address.
- Diagnosis of CD or UC if already known
- Status of current treatment in known IBD patients [22, 24, 25, 45] (EL 1a–2b)
  Therapy status is an important piece of information to provide as pathologists can then determine whether treatment has achieved the desired endpoint of histologic mucosal healing. For example, if a pathologist with access to previous biopsy findings and therapy information encounters normal mucosa in areas of known, formerly active IBD, they can establish in their report that histologic normalization has occurred, to indicate that the mucosa has, in fact, normalized due to treatment.
- Specific, designated sites from which biopsies have been taken [45, 55].
  Biopsies from different bowel segments should be submitted in such a way that their site of origin can be determined reliably. This issue is discussed further with statement 2, mentioned previously.

Core histologic features to be communicated to the clinicians include:

- Diagnosis of IBD or others
- Distinction between UC and CD [16, 44–51, 102] (EL 1b–2a)
- The term “indeterminate colitis” should be avoided as it causes confusion and can be used incorrectly. Rather, it is recommended that a pathologist state that IBD is present and the features favour one disease type over the other, but that definitive classification may depend on the clinical course.
- Exclusion of dysplasia or malignancy [62, 64]
- Information regarding the adequacy and/or limitations of the submitted material, i.e. a statement about the sufficient number of biopsies (see recommendation 2)
  - In case of insufficiently labelled containers, feedback to the clinicians that the origin of the biopsies cannot reliably be assessed, limiting assessment for IBD diagnosis.
  - Potential limitations due to biopsy orientation or artefacts
  Assessment of certain features such as distribution of changes or presence and extent of a basal plasmacytosis is limited if the material is insufficient and poorly oriented. Use of acetate strips can be helpful for proper biopsy orientation [45, 103].

#### Recommendation 6

*The histological assessment should evaluate the distribution of overall findings, presence of chronic injury and activity of inflammation (vote: strong consensus (100%), level of evidence: 1a–3b, recommendation grade: C).*

### Evidence and decision

#### Distribution

Evaluating the distribution of microscopic changes can serve as a quantitative marker for assessing overall deviations from normal mucosa. It particularly pertains to the pattern and amount of tissue involved by chronic injury and active inflammation. The abnormalities can vary within a single biopsy, within different biopsies from the same site as well as among different anatomical sites. This heterogeneity of disease involvement is typical of CD but can also be seen in UC especially after treatment [60, 66–73, 75, 76, 78–91, 93] (EL 1b–2b).

Terms that are sometimes used by pathologists to assess disease involvement, such as “diffuse”, “patchy” or “focal”, are ill-defined and can be subjective. It is preferable to use alternative terms such as “continous between sites”, “discontinous between sites” or “segmental” (anatomically non-continuous) [14] (EL 2a). Assessing the distribution of changes is helpful for distinguishing UC from CD. Moreover, in the course of known IBD, reporting the distribution of changes can provide important information regarding response to treatment in a quantitative fashion [4, 60–62, 74, 104, 105] (EL 1a–2a).

#### Chronicity

As most adult IBD patients present after at least 6 weeks of symptoms, features of chronic injury are often already discernible on their GI biopsies at initial diagnosis [10, 106] (EL 2a–2b). Features of chronic injury include architectural distortion and chronic inflammatory infiltrates in the lamina propria [10] (EL 2a). Basal plasmacytosis is probably the earliest sign of chronic injury and has proven to be the strongest and highest reliable predictor of IBD. This feature was demonstrated to achieve almost perfect intrarater as well as substantial interrater reproducibility rates and can have significant prognostic implications. Moreover, on rectal biopsy specimens, basal plasmacytosis has been shown to be one of several independent predictors of earlier relapse in UC patients [9, 10, 21, 29, 107, 108] (EL 2a–3b). Another feature of chronic injury, architectural distortion, is typically not seen before 15 days of symptoms. However, it is present in more than 75% of patients after 4 months [10, 16] (EL 2a). This feature is also associated with almost perfect intraobserver and substantial interobserver agreement rates [107] (EL 2b). Some of the existing scoring indices for evaluating UC disease activity do not include architectural distortion, as this feature was not considered to be a marker for disease activity and was also considered unlikely to be responsive to therapy. This, however, proved to be false, as complete histologic mucosal normalization is now an attainable treatment goal [10, 37, 104, 109] (EL 1b–2b). Crypt architectural distortion may, in fact, be the only feature able to distinguish between remission and true histological normalization, given the feature’s lengthier persistence. This is in contrast to basal plasmacytosis, which may diminish earlier with longstanding or treated disease [10, 45, 110] (EL 2a–3b). Additional features of chronic injury are Paneth cell metaplasia (especially distal the splenic flexure), pseudopolyps, hypertrophy of the muscularis mucosae and submucosal fibrosis [105] (EL 2a).

The role of increased eosinophils in IBD is unclear. Eosinophils are part of the normal mucosa, and their number is highly variable [93] (EL 2a). Due to a lack of reproducibility and only moderate interobserver agreement, the ECCO recommended that eosinophils alone should not be used as a marker of histological activity in UC [16, 41, 106] (EL 2a).

#### Activity

Disease activity as measured histologically is ascertained by the presence of neutrophilic granulocytes and their damage to the epithelium. Cryptitis, crypt abscess formation as well as erosions and ulcerations represent the spectrum of active inflammation in order of increasing severity [11, 12, 14, 111, 112] (EL 1a–2b). Whilst some question the significance and reproducibility of histologic activity, the presence of acute inflammatory infiltrates is actually associated with a twofold to threefold increased risk of relapse within 12 months of follow-up and an increased use of systemic corticosteroids, colectomy and hospitalization within 3 years of follow-up [20, 113] (EL 2b). Moreover, assessing for acute inflammatory infiltrates such as neutrophils in the lamina propria, intraepithelial neutrophils, erosions or ulceration attained good reproducibility in existing scoring systems [107, 109, 114–116] (EL 2b).

##### Recommendation 7

*The scoring system (the IBD-DCA score) developed by the consensus group based on the factors of distribution (D) of changes, chronic injury (C) and activity of inflammation (A) is a proposal for a standardized and user-friendly tool for histologic activity assessment (vote: strong consensus (98%), level of evidence: 2b, recommendation grade: B).*

The proposed “Inflammatory Bowel Disease – Distribution, Chronicity, Activity” (IBD-DCA) score comprises the three parameters—distribution (D), chronicity (C) and activity (A)—and is determined in that order. The individual items constituting the parameters C and A were selected on the basis of previous research, with particular selection of features that have achieved good reliability in existing scoring systems in order to be eligible candidates for the index development (supplemental Table 2).

#### Distribution parameter (D)

Parameter D is used to estimate the overall extent of mucosal abnormalities, regardless of whether they represent architectural distortion, chronic inflammation or active inflammatory infiltrates. D0 is assigned for completely normal mucosa without any pathological changes. D1 is assigned if changes are present in less than 50% of all tissue fragments. D2 is assigned for changes that involve 50% or more of the tissue.

#### Chronicity parameter (C)

Parameter C encompasses crypt architectural distortion as well as elevated lymphoplasmacytic cell count in the lamina propria (including basal plasmacytosis). C0 refers to mucosa without chronic changes, i.e. absence of elevated lymphoplasmacytic cell count and absence of crypt architectural distortion. C1 refers to crypt distortion and/or mildly elevated lamina propria lymphoplasmacytosis (mildly more lymphocytes and plasma cells than in the normal mucosa). C2 necessitates a marked elevated lymphoplasmacytic cell count in the lamina propria regardless of the additional presence of crypt distortion. A marked basal lymphoplasmacytosis is also assessed as C2.

#### Activity parameter (A)

Parameter A refers to the presence and degree of tissue involvement by neutrophilic granulocytes. A0 is assessed in the absence of neutrophilic granulocytes. The number of neutrophils that are allowed in a normal lamina propria ranges in the literature between 0 and 1 [17, 93] (EL 2a). Neutrophils are not normally present in the surface or crypt epithelium [93] (EL 2a). Therefore, A1 is assessed for an increase of two or more neutrophils in the lamina propria in one high-power field (HPF) or one or more neutrophils in the epithelium (as in cryptitis or neutrophils in the surface epithelium). To reach the intestinal lumen, neutrophils must first exit the blood vessels, migrate across the lamina propria and finally cross the epithelial barrier in that order [77] (EL 2a). Therefore, A2 is assigned in the presence of crypt abscesses, erosion or ulceration as they have breaks of the mucosal barrier in common.

A validation of the IBD-DCA score was performed after the meeting (Lang-Schwarz et al., submitted).

Figure 1 shows the proposed algorithmic assessment of the IBD-DCA score.

##### Recommendation 8

*The pathology report can include routine text as well as the IBD-DCA score as qualitative and quantitative information for clinicians (vote: consensus (93%), level of evidence: 5, recommendation grade: D).*

##### Evidence and decision

The IBD-DCA score is not intended to replace the pathologist’s individual text reports, but it can act as an additional tool to summarize histologic activity qualitatively and quantitatively. Clinicians and pathologists in their professional partnerships may already have established means of relaying important patient information with one another through the language of their individual reports. The additional proposed benefit in reporting the IBD-DCA score, however, is its facilitation in disease monitoring, given that each parameter can be quickly and easily compared to previous findings during a patient’s follow-up.

Example reports:

- Ulcerative proctitis, marked activity; IBD-DCA score: D2, C2, A2
- Ulcerative proctitis, remission; IBD-DCA score: D1, C1, A0
- Ulcerative proctitis, histologic mucosal healing; IBD-DCA-score: D0, C0, A0

##### Recommendation 9

*Activity scoring is ideally performed for every container separately (vote: consensus (83%), level of evidence: 1b–3b, recommendation grade: C).*

##### Evidence and decision

Scoring each site separately may help distinguish CD from UC diagnostically. A discontinuous and focal pattern of disease involvement is typical for CD, whereas UC classically shows diffuse mucosal inflammation [4, 108] (EL 2a–3b). There is also a known gradient of disease severity that increases from proximal to distal colon in UC, versus the opposite pattern in CD in many cases, which is helpful in distinguishing both entities [4] (EL 2a). Performing the above scoring index for each gastrointestinal biopsy site can, in fact, highlight these distinguishing attributes and support the diagnosis. Secondly, as treatment is able to alter the usual distribution of IBD and therapy response can vary between bowel segments, the participants agreed that activity scoring according to the IBD-DCA score should be performed for each designated site [61, 66–70] (EL 1b–2b). Nevertheless, effective scoring obviously depends on the endoscopist’s sampling, as detailed in statements above. Moreover, scoring and reporting each site if diagnosis and result are exactly the same for every container may not be necessary and a representative single score encompassing those parts could be done in such situations.

- Example: ulcerative pancolitis, markedly active in sigmoid (D2C2A2), mildly active in all other sites (D2C1A1)

##### Recommendation 10

*The IBD-DCA score is appropriate for use in daily routine practice (vote: strong consensus (95%), level of evidence: 5, recommendation grade: D).*

##### Evidence and decision

The design of the IBD-DCA score is intended to be simple and user-friendly, with the purpose of emulating the manner in which pathologists intuitively assess samples from low to high power magnification in daily practice. Therefore, it is well suited to be applied to biopsy specimen in daily routine diagnostics as well as clinical trials. It can be applied to biopsies from UC as well as CD patients. However, its significance in CD and also its applicability to the upper GI tract have to be further investigated. The validation of the index has proven its reproducibility, feasibility, construct validity as well as its ability to provide reliable information about response to treatment (Lang-Schwarz et al., submitted).

## Conclusion and further perspectives

Assessing the presence and degree of disease activity in IBD patients is important for developing optimal therapeutic strategies and timing of follow-up [117–119]. As UC activity scoring warrants both endoscopic and histologic evaluations, there is a need for standardized biopsy procedures and a simple, validated scoring system that can be used globally. Moreover, such a scoring index should be able to discriminate between quiescence and histologic mucosal healing as histologic mucosal healing is emerging as the desired therapeutic goal. Histological mucosal healing is not well defined to date. In their “Histology Position 3.4”, the ECCO refers to “return to normal” as the strictest definition of histological remission [41]. A new key feature of histologic mucosal healing might therefore lie in identifying the absence of crypt architectural distortion, thus distinguishing between quiescent UC (which has architectural distortion) and true histological normalization (which looks like normal colon) [10, 74]. Two recent studies by Christensen et al. [37, 120] suggest that histologic normalization differs clinically from histologic quiescence by being independently associated with increased relapse-free survival in UC and CD, reduced medication escalation and reduced corticosteroid use [37, 120]. To date, many different scoring systems exist for assessing histologic activity in UC and CD. The Cochrane Collaboration recently reviewed 14 different histologic scoring systems for CD as well as 30 indices for UC by highlighting their different advantages and disadvantages [39, 40]. Some of the systems are simple, whilst others are very detailed. For UC, the Nancy Histological Index (NHI) and the Robarts Histopathology Index (RHI) have undergone the most validation, even if the feasibility of these indices has not been assessed [109, 114]. Therefore, the ECCO recommended recently the use of both indices for randomized control trials in ulcerative colitis and the use of the NHI for observational studies or in clinical practice [41].

However, neither the NHI nor the RHI includes architectural features, which means that they cannot distinguish between histologic quiescent disease (in which crypt architectural distortion is often the only visible feature) and histological complete normal mucosa. As for CD, the existing indices have yet to be validated and none of them is recommended for use to date.

In order to address such issues, the International Consensus Conference on IBD Activity Scoring assented on the above statements with the goal of maximizing diagnostic information from biopsies as relevant in the modern era of IBD therapy and of optimizing communication between clinicians and pathologists to enhance patient care. The statements are in line with published recommendations from internationally recognized organizations such as ECCO, the BSG, the ACG, the ASGE and the WGO, and they are intended to consolidate the factors most relevant to patient care and raise awareness regarding the need for these clinically relevant matters to be applied more consistently in daily practice. The group agreed on ten statements which received a high level of agreement. Since the participants represented centres from different countries worldwide, this agreement suggests notable international recognition on the topics considered.

Additionally, we present the IBD-DCA score, a proposal for an international, consensus-approved histologic activity scoring index, intended for use in routine practice and clinical trials. The scoring index is able to distinguish between quiescence and true histological mucosal healing. Furthermore, it includes a statement on the quantity as well as the quality of inflammatory changes. Apart from normal, grading of chronicity and activity is reported in a two-tiered fashion in the IBD-DCA score, distinguishing only between low- and high-level inflammatory changes. The terms “low-” and “high-grade” are, however, intentionally omitted to avoid confusion as these terms are traditionally used for dysplasia. The simplicity of the index makes it likely to be used by pathologists and also to be accepted by the clinicians. It should be applicable for UC and may also be applicable for CD in both the upper and lower GI tract. Noteworthy that the index may be used for patients with proven IBD during follow-up to assess changes over time. However, the main limitation of this study is the fact that the proposed index has not been tested prospectively so far. Validation by an independent research group is clearly necessary. Also, its use in CD needs further validation studies, due to the potential limitations of histologic activity scoring in the discontinuous disease involvement inherent in the nature of CD.

## Supplementary Information

Supplemental Fig. 1 (PNG 245 kb)

High resolution image (TIF 83 kb)

Supplemental Table 1 (DOCX 20 kb)

Supplemental Table 2 (DOCX 15 kb)

## Data Availability

Data and material (as far as not published) are available on demand via e-mail at vieth.lk-pathol@uni-bayreuth.de.
