# Supplementary figures and images for: Maximizing the diagnostic information from biopsies in chronic inflammatory bowel diseases: recommendations from the Erlangen International Consensus Conference on Inflammatory Bowel Diseases and presentation of the IBD-DCA score as a proposal for a new index for histologic activity assessment in ulcerative colitis and Crohn’s disease
Source: Virchows Arch. 2020 Dec 29;478(3):581–94. doi: 10.1007/s00428-020-02982-7 (PMC7973393; doi:10.1007/s00428-020-02982-7)

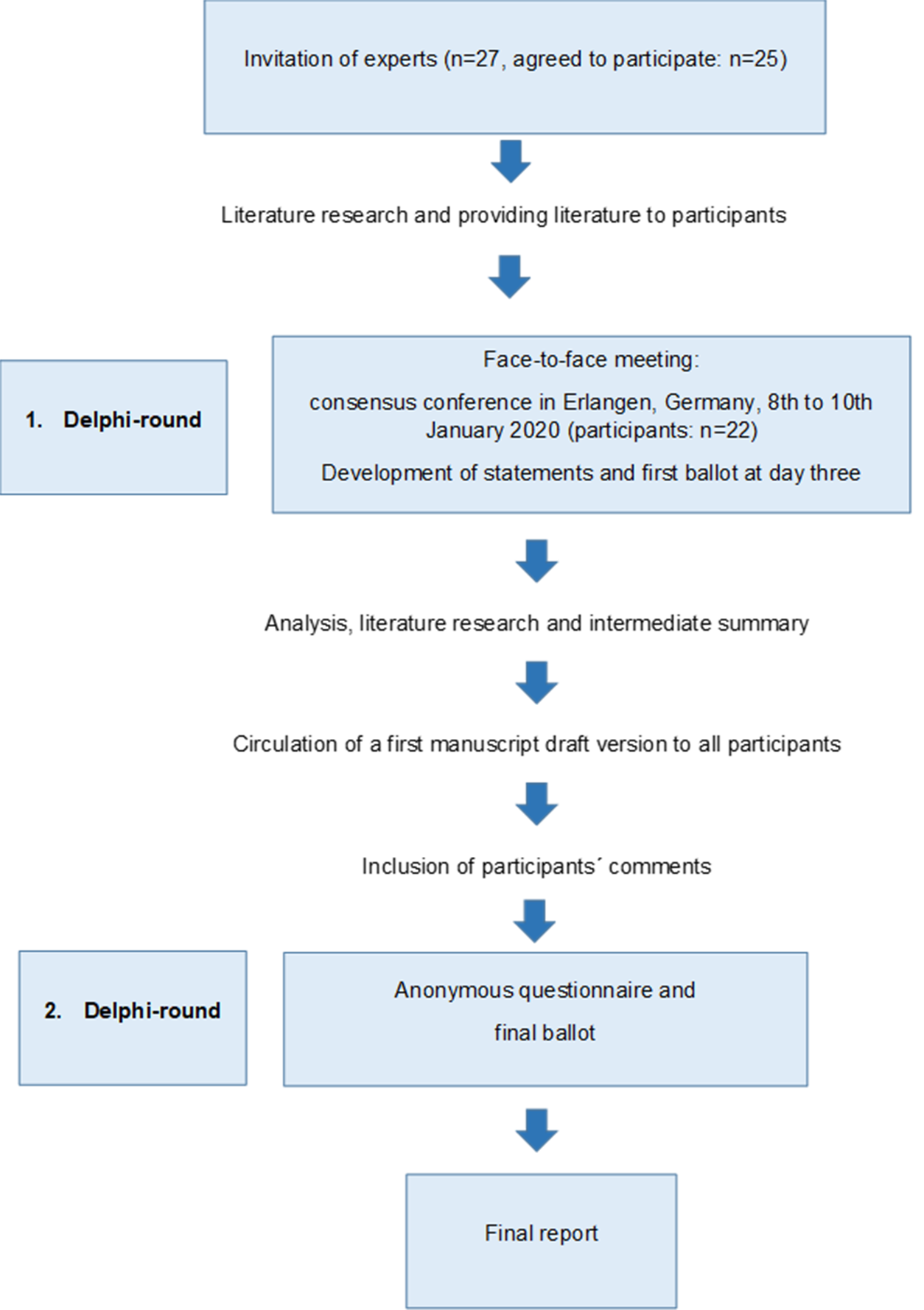

Supplement: Supplementary file 1 — (PNG 245 kb) [file 428_2020_2982_Fig2_ESM.png]

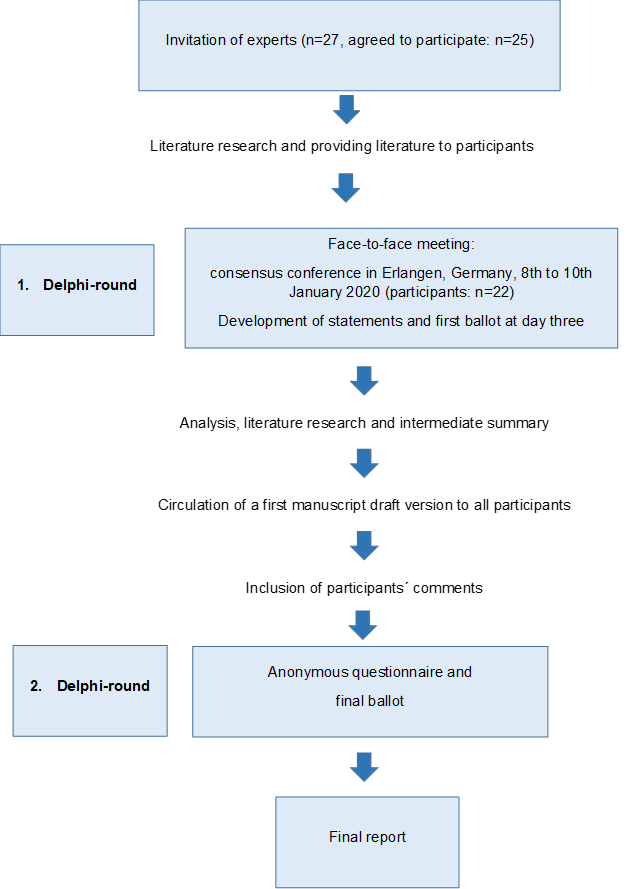

Supplement: Supplementary file 2 — High resolution image (TIF 83 kb) [file 428_2020_2982_MOESM1_ESM.tif]
