# Supplementary material for: Maximizing the diagnostic information from biopsies in chronic inflammatory bowel diseases: recommendations from the Erlangen International Consensus Conference on Inflammatory Bowel Diseases and presentation of the IBD-DCA score as a proposal for a new index for histologic activity assessment in ulcerative colitis and Crohn’s disease
Source: Virchows Arch. 2020 Dec 29;478(3):581–94. doi: 10.1007/s00428-020-02982-7 (PMC7973393; doi:10.1007/s00428-020-02982-7)
Supplement: Supplementary file 3 — (DOCX 20 kb) [file 428_2020_2982_MOESM2_ESM.docx]

| **Reference, first author** | **Study type** | **Nr. of cases** | **Nr. of observers** | **Level of evidence according CEBM** | **Outcome, Aims or Findings** |
| --- | --- | --- | --- | --- | --- |
| 2, Kucharzik A | S3 Guideline, German | --- |  | --- | --- |
| 3, Allison MC | Correlation study, retrospective | 72 | 2 | 2a | Distinction criteria between idiopathic colitis and IBD |
| 8, Jenkins D | Guideline, British Society of Gastroenterology | --- | --- | --- | --- |
| 9, Nostrant TT | Prospective, case control study | 48, 36, 84 | 2 | 2a | Distinction criteria between idiopathic colitis and IBD |
| 10, Schumacher G | Prospective case control study, long term follow up | 105 |  | 2a | IBD versus other colitis |
| 11, Seldenrijk CA | Prospective, blinded, case control study | 61 (45+16 controls) | 4 | 1a | IBD versus other colitis |
| 12, Surawicz CM | Retrospective, blinded | 148 |  | 2b | IBD versus self-limiting colitis |
| 14, Tanaka M | Retrospective, large cases series | 299+132 |  | 2a | criteria distinguishing IBD from other forms of colitis, and CD from UC |
| 16, Theodossi A | Retrospective case series | 76 | 10 | 2a | Discriminatory morphological features |
| 17, Langer C | Practice guide | --- | --- | --- | --- |
| 20, Riley SA | Prospective, 12 months follow up | 82 | 2 | 2b | Clinical relapse |
| 21, Bitton A | Prospective clinical trial, max. 12 months follow up | 74 |  | 1a | Clinical relapse |
| 22, Neurath MF | Systematic review | --- | --- | 1a | --- |
| 24, Froslie KF | Large retrospective cohort study with 5 years follow up, endoscopic | 740, 495 with follow up | --- | 2b | Possible predictors of mucosal healing, impact of healing on subsequent course of disease. |
| 25, Colombel JF | multicenter, randomized, double-blind, placebo-controlled study, conducted globally | Intitial 728, last follow up after 54 weeks 121 | multicenter | 1b | Association between early improvement and clinical outcome |
| 29, Bessissow T | Retrospective, 12 month follow up | 75 | 1 | 2b | Clinical relapse |
| 34, Bryant RV | Blinded, 6 years follow up, prospective | 91 | 1 | 1b | Clinical relapse, hospitalisation, corticosteroids |
| 38 Christensen | Retrospective case-control-study | 646 | 2 | 2b | Clinical relapse |
| 42, Marchal-Bressenot A | Retrospective, partly validated | 200, 100 | 3 | 2b | Index development and validation, responsiveness |
| 43, Mosli MH | Study cohort, retrospective, partly validated | 48 | 4 | 2b | Index development and validation |
| 47, Annese V | European Evidence based Consensus | --- | --- | --- | --- |
| 52, Bernstein C | Guideline | --- | --- | --- | --- |
| 53, Magro F | ECCO Consensus IBD, 2013 | --- | --- | --- | --- |
| 54, Dignass A | Evidence-based Consensus, UC | --- | --- | --- | --- |
| 55, Lamb CA | BSG, Consensus Guidelines on IBD | --- | --- | --- | --- |
| 56, Sharaf RN | ASGE Guideline on Tissue Sampling | --- | --- | --- | --- |
| 61, Coremans G | Prospective | 110 |  | 2a | Diagnostic value of ileoscopy and biopsy |
| 62, Carbonnel F | Retrospective cohort analysis | 85 | 1 | 2b | Value of colonoscopy for the assessment of colonic ulcerations |
| 63, Alemayehu G | Small cohort study, prospective, median follow up 5 years | 34 | --- | 2b | Value of colonoscopy during severe attack of ulcerative colitis |
| 48, Gomollon F | European Evidence based Consensus | --- | --- | --- | --- |
| 37, Kleer CG | retrospective | 41 patients, 217 biopsies | --- | --- | Time related endoscopic and histologic findings in UC and CD |
| 64, Scott IS | retrospective | 142 | --- | 2b | Prevalence and histological features of appendiceal inflammation in patients with UC and CD |
| 65, Odze R | Review article | --- | --- | 1a | --- |
| 66, D´Haens G | Prospective, small number of patients | 20 | --- | 2b | Extent and continuity of mucosal inflammation incidence of skip lesions in UC |
| 67, Kroft SH | Retrospective study | 39 |  | 2b | Distribution patterns of UC |
| 59, Mutinga ML | Retrospective cohort study with control, mean follow-up for study and control groups 105 +/- 128 and 112 +/- 80 months | 12 out of 352 |  | 2a | Clinical course |
| 68, Bernstein CN | Prospective | 39 | --- | 2b | Distribution of changes in UC |
| 60, Geboes K | Review | --- | --- | 2a | --- |
| 70, Markowitz J | Blinded, retrospective | 12+62 | --- | 2b | Role of rectosigmoid histology in IBD |
| 71, Robert ME | Blinded comparative study, min 1 year follow up | 15+25 | --- | 1b | Differences between adults and children with UC |
| 72, Tanaka M | Review | --- | --- | 2a | --- |
| 73, Marshall JK | Systematic review | 38 studies included | --- | 1a | --- |
| 74, Lie MR | Systematic review and metaanalysis | 23 studies included | --- | 1a | --- |
| 75, Cohen RD | Systematic review | 48 publications included | --- | 1a | --- |
| 76, Mosli MH | Systematic review | 18 indices included | --- | 1a | --- |
| 77, Odze R | Prospective, placebo controlled trial | 14 patients, 123 biopsies | --- | 1b | 5ASA versus placebo – comparism of histology in UC patients |
| 78, Billioud V | Review, metaanalysis | 21 studies | --- | 1a | --- |
| 79, Herfarth H | double blind, double dummy, randomised, prospective, multicentre study | 79 | --- | 1a | severe endoscopic relapse, clinical relapse, adverse drug reaction |
| 80, D´Haens GR | Randomized controlled trial | 81 | --- | 1b | Clinical relapse |
| 81, Nos P | Prospective study, 2 years follow up | 34 | --- | 1b | Clinical relapse |
| 82, Reinisch W | Randomized controlled trial, double-blind, double-dummy, multicentered | 78 | --- | 1b | Clinical relapse |
| 83, Peyrin-Biroulet L | Systematic review | --- | 2 | 2a | --- |
| 84, Regueiro M | Randomized controlled trial, 1 year follow up | 24 | --- | 1b | Postoperative recurrence |
| 85, Regueiro M | Prospective study, follow up 4 years | 24 | --- | 1b | Clinical relapse |
| 86, Yoshida K | Clinical trial with control arm, randomised, 36 months follow up | 31 | --- | 1b | Clinical relapse |
| 87, Greuter T | Swiss IBD cohort | 1638 | --- | 2b | Analysis of risk factors and disease course of patients with upper GI tract involvement in CD |
| 88, Castellaneta SP | Prospective cohort study, pediatric | 65 | --- | 2b | Importance of upper GIT endoscopy in diagnosis IBD and assessment of factors predictive of upper GIT involvement in IBD |
| 89, Lin J | Retrospective cohort study | 69 | --- | 2b | Role of upper GIT involvement in UC |
| 90 Oberhuber G | Prospective, comparative study | 75 + 200 controls | 2 | 1b | Histological appearance of gastric mucosa in patients with CD |
| 49, Feakins RM | Practice guideline | --- | --- | --- | --- |
| 92, Levine DS | Review | --- | --- | 2a | --- |
| 50, Stange EF | ECCO Consensus UC, 2008 | --- | --- | --- | --- |
| 51, Stange EF | ECCO Consensus CD, 2006 | --- | --- | --- | --- |
| 57, Carter MJ | Guidelines, British Society of Gastroenterology | --- | --- | --- | --- |
| 93, Cosnes J | Systematic review | --- | --- | 2a | --- |
| 94, Molodecky NA | Systematic review | 260 studies | --- | 1b | --- |
| 95, Burisch J | Prospective, uniformly diagnosed, population based inception cohort study, multicentered | 10.1 mio | --- | 1a | --- |
| 96, Lakatos L | Hungarian cohort study, retrospective, 5 year follow up | 393 | --- | 2a | --- |
| 97, Shivananda S | Prospective multicentered comparative study | 2201 | --- | 1a | Higher overall incidence rates in northern centres but recent increases in the incidence of IBD in southern Europe |
| 98, Björnsson S | Prospective epidemilogic study, Iceland | 215 | --- | 1b | Continuing and statistically significant increase in the incidence of both UC and CD in Iceland |
| 99, Vind L | Danish population based study, prospective, follow up 1-3 years | 562 | --- | 1b | Incidence of IBD increasing, time until diagnosis decreasing |
| 100, Langholz E | Retrospective cohort analysis, Copenhagen | 1161 | --- | 2b | Prevalence of UC increasing |
| 101, Moum B | Prospective population based study, norway, follow up 3-6 years | 525 | --- | 1b | High relapse rate for UC and CD |
| 70, Geboes K | review |  | --- | 1b | --- |
| 58, Feakins RM | Practical guideline | --- | --- | --- | --- |
| 103, Mosli MH | Retrospective | 49 | 5 experts | 2b | Reproducibility assessment of histological activity indices |
| 102, Geboes K | Prospective case control study with follow up | 257 vs 53 controls |  | 1b | --- |
| 104, Kim B | Prospective cohort study with follow up | 32 | --- | 1b | No significant difference in treatment, between those with patchiness and/or rectal sparing and those without |
| 105, Rubio CA | Case control study | 61 UC, 124 controls |  | 3b | Features to differentiate between UC remission patients and non-colitic patients |
| 106, Gramlich T | Review |  |  | 2a | IBD in children |
| 107, Pai RK | Review |  |  | 2a | Assessment of disease activity in UC |
| 108, Sadik CD | Review |  |  | 2a | Role of neutrophils in inflammation |
| 109, Chin AC | Review |  |  | 2a | Pathobiology of neutrophil transepithelial migration |
| 110, Pai RK | Retrospective, 3 years follow up, single-center study | 889 biopsies from 281 patients with UC |  | 2b | Correlations between histologic features and clinical outcomes |
| 111, Feagan BG | multicenter, double-blind, placebo-controlled clinical trial | 181 |  | 1b | Clinical and endoscopic remission |
| 112, Surawicz CM | Blinded retrospective comparative study | 52 non IBD, 81 IBD |  | 3b | Rectal biopsy specimens are useful in distinguishing ASLC from acute-onset IBD |
| 113, Washington K | Multicenter study, pediatric, retrospective, blinded | 53 children, 38 adults |  | 2b | Differences between UC in adults versus children |
| 114, Bressenot A | Comparative study, retrospective | 102 biopsies | 3 | 2b | Strong correlation between histological indices |
| 116, Fournier BM | Review |  |  | 2a | The role of neutrophils during intestinal inflammation |
| 120, Christensen B | Retrospective, single center | 101 CD patients | 2 pathologists | 2b | Comparism of outcomes between histologic and endoscopic healing in CD |
