# Supplementary material for: Maximizing the diagnostic information from biopsies in chronic inflammatory bowel diseases: recommendations from the Erlangen International Consensus Conference on Inflammatory Bowel Diseases and presentation of the IBD-DCA score as a proposal for a new index for histologic activity assessment in ulcerative colitis and Crohn’s disease
Source: Virchows Arch. 2020 Dec 29;478(3):581–94. doi: 10.1007/s00428-020-02982-7 (PMC7973393; doi:10.1007/s00428-020-02982-7)
Supplement: Supplementary file 4 — (DOCX 15 kb) [file 428_2020_2982_MOESM3_ESM.docx]

| **Item** | **ICC (95% CI)** | **References** | **Corresponding parameter in IBD-DCA score** |
| --- | --- | --- | --- |
| Chronic inflammatory infiltrate | 0.750 (0.640-1)  0.75 (0.54-0.86) | NHI [1]  RHI [2] | C |
| Basal plasmacytosis | 0.63 (0.48-0.74) | Mosli et al [3] | C 1 and C 2 |
| Crypt architectural distortion | 0.72 (0.59-0.80)  0.70 (0.56-0.79) | Mosli et al for MRS and for GS [3-5] | C 1 |
| Acute inflammatory infiltrate | 0.772 (0.704-0.940)  0.85 (0.82-0.88) | NHI [1]  Bressenot et al for RI [6, 7] | A 1 |
| Lamina propria neutrophils | 0.61 (0.48-0.69)  0.82 (0.78-0.86) | Mosli et al and Bressenot et al for GS [2, 5, 7] | A 1 |
| Neutrophils in epithelium | 0.74 (0.68-0.80) | Bressenot et al for GS [5, 7] | A 1 |
| Erosion | 0.79 (0.66-0.86)  0.82 (0.77-0.88) | RHI [2]  Bressenot et al for  GS [5, 7] | A 2 |
| Ulceration | 0.865 (0.750-1)  0.79 (0.66-0.86)  0.82 (0.77-0.88)  0.90 (0.79-0.97) | NHI [1]  RHI [2]  Bressenot et al for GS and for Gramlich Index [5, 7, 8] | A 2 |

Abbreviations: ICC, Intraclass Correlation Coefficient; CI, Confidence Interval; NHI, Nancy Histological Index; RHI, Robarts histopathology index; MRS, Modified Riley Score; GS, Geboes Score; RI, Riley Index.

References:

1. Marchal-Bressenot A, Salleron J, Boulagnon-Rombi C, et al (2017) Development and validation of the Nancy histological index for UC. Gut 66:43-49. https://doi.org/10.1136/gutjnl-2015-310187
2. Mosli MH, Feagan BG, Zou G et al. Development and validation of a histological index for UC. *Gut*2017;66:50-58 doi:10.1136/gutjnl-2015-310393 [published Online First: 16 October 2015].
3. Mosli MH, Feagan BG, Zou G, et al. Reproducibility of histological assessment of disease activity in UC. *Gut*2014;0:1-9 doi:10.1136/gutjnl-2014-307536 [published Online First: 30 October 2014].
4. Feagan BG, Greenberg GR, Wild G, et al. Treatment of ulcerative colitis with a humanized antibody to the alpha4beta7 integrin. *N Engl J Med*2005;352:2499-507 doi:10.1056/NEJMoa042982.
5. Geboes K, Riddell R, Ost A, et al. A reproducible grading scale for histological assessment of inflammation in ulcerative colitis. *Gut*2000;47:404-9 doi:10.1136/gut.47.3.404. .
6. Riley SA, Mani V, Goodman MJ, et al. Microscopic activity in ulcerative colitis: what does it mean? *Gut*1991;32:174–178 doi:10.1136/gut.32.2.174.
7. Bressenot A, Salleron J, Bastien C, et al. Comparing histological activity indexes in UC. *Gut*2015;64(9):1412-8 doi:10.1136/gutjnl-2014-307477 [published Online First: 22 September 2014].
8. Gramlich T, Petras RE. Pathology of inflammatory bowel disease. *Semin Pediatr Surg* 2007;16;154-63 doi:10.1053/j.sempedsurg.2007.04.005.
